# Sex-specific chronic stress response at the level of adrenal gland modifies sexual hormone and leptin receptors

**DOI:** 10.3325/cmj.2015.56.104

**Published:** 2015-04

**Authors:** Marta Balog, Milan Miljanović, Senka Blažetić, Irena Labak, Vedrana Ivić, Barbara Viljetić, Attila Borbely, Zoltán Papp, Robert Blažeković, Sandor G. Vari, Miklós Fagyas, Marija Heffer

**Affiliations:** 1 J. J. Strossmayer University of Osijek, Faculty of Medicine, Osijek, Croatia; 2 J. J. Strossmayer University of Osijek, Department of Biology, Osijek, Croatia; 3 University of Debrecen, Faculty of Medicine, Institute of Cardiology, Debrecen, Hungary; 4 Department of Cardiac and Transplantation Surgery, University Hospital Dubrava, Zagreb, Croatia; 5 International Research and Innovation Management Program, Cedars-Sinai Medical Center, Los Angeles, CA, USA; * Equally contributing authors.

## Abstract

**Aim:**

To compare cardiometabolic risk-related biochemical markers and sexual hormone and leptin receptors in the adrenal gland of rat males, non-ovariectomized females (NON-OVX), and ovariectomized females (OVX) under chronic stress.

**Methods:**

Forty six 16-week-old Sprague-Dawley rats were divided into male, NON-OVX, and OVX group and exposed to chronic stress or kept as controls. Weight, glucose tolerance test (GTT), serum concentration of glucose, and cholesterol were measured. Adrenal glands were collected at the age of 28 weeks and immunohistochemical staining against estrogen beta (ERβ), progesterone (PR), testosterone (AR), and leptin (Ob-R) receptors was performed.

**Results:**

Body weight, GTT, serum cholesterol, and glucose changed in response to stress as expected and validated the applied stress protocol. Stressed males had significantly higher number of ERβ receptors in comparison to control group (*P* = 0.028). Stressed NON-OVX group had significantly decreased AR in comparison to control group (*P* = 0.007). The levels of PR did not change in any consistent pattern. The levels of Ob-R increased upon stress in all groups, but the significant difference was reached only in the case of stressed OVX group compared to control (*P* = 0.033).

**Conclusion:**

Chronic stress response was sex specific. OVX females had similar biochemical parameters as males. Changes upon chronic stress in adrenal gland were related to a decrease in testosterone receptor in females and increase in estrogen receptor in males.

Maintaining homeostasis is often challenged by different types of stressors (1). Homeostasis is regulated by a complex endocrine processes engaging the hypothalamic-pituitary-adrenal axis (HPA) and sympathetic autonomic system (2-4). Stress can occur either in acute or chronic form with different consequences – the acute stress mostly induces the ˝fight or flight˝ response, while chronic stress promotes long term changes, which can lead to a variety of diseases (5,6). If stress is of sufficient magnitude and duration, the action of HPA is unsuppressed and results in prolonged elevation of cortisol (7), induced production of energy, vasoconstriction, lipolysis, proteolysis, immunosuppression, and suppression of reproductive function to save energy and retain overall homeostasis (8). Women are generally less susceptible to chronic stress up to the period of menopause, when the loss of protective hormones, estrogen and progesterone, occurs and thus they become prone to development of depression, anxiety, or schizophrenia (9). In contrast, men are generally more susceptible and sensitive to chronic stress, showing changes in feeding habits and decreased body weight (10,11).

Chronic stress can cause the development of cardiovascular disorder, obesity, and diabetes, which can be reflected in serum cholesterol, glucose, and decreased glucose tolerance (12-14). There is a strong correlation between stress and sexual hormones, but the mechanisms by which estrogen, testosterone, and progesterone exert their possible protective role under stress conditions are not fully explored. Sexual hormones affect stress outcome and stress hormones affect the levels of sexual hormones (15-17). Testosterone is activated during stress response in rats and humans (18,19) and tends to increase more in men than women (20). Estrogen lowers the stress-induced response in women and men (9,21). Estrogens and progesterone are produced even after ovariectomy by adrenal glands (22) but it is not known if such compensation can withstand additional challenge like stress. Another possible player in stress response is leptin (Ob), hormone responsible for maintaining body weight, which is synthesized and secreted by adipose tissue (23), exerting its effects through the leptin receptor (Ob-R) (24). Chronic stress models imply a direct link between stress response and leptin (25,26). Receptors for leptin are present in the adrenal gland (27). The aim of this study was to investigate cardiovascular risk parameters and changes in leptin and sexual hormone receptors in adrenal gland during chronic stress. There is a clinically relevant change in the onset of cardiometabolic risk between healthy women and women with premature ovarian failure (28) and because of that ovariectomized female rats were included in the study.

## Materials and methods

### Animals

**Figure 1:**
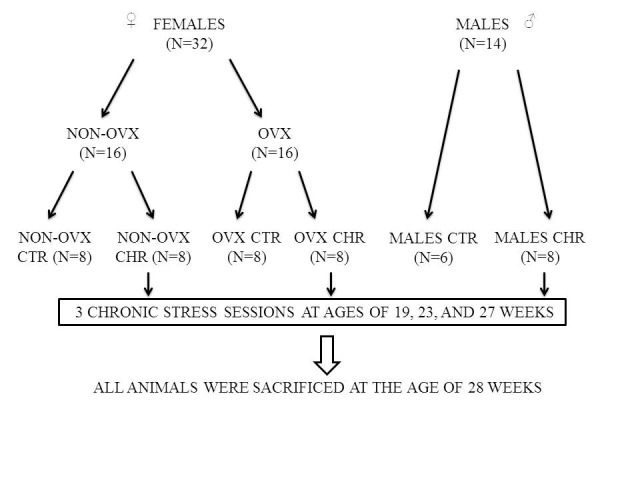
Flowchart presenting the animal groups and the stress protocol timeline. CTRL = control, OVX = ovariectomized, NON-OVX = non ovariectomized.

The study was conducted during 2013 and 2014 at the Faculty of Medicine Osijek and was approved by the corresponding ethics committees. Forty six 16-week-old Sprague-Dawley rats (22 males and 24 females) were divided in three groups: males, non-ovariectomized (NON-OVX) females, and ovariectomized (OVX) females. Each animal group was further divided into chronic stress and control group (Figure 1). Every group consisted of 8 animals, except the male control group, which consisted of 6 animals. Animals were housed in standard cages at room temperature. Standard laboratory rat food and tap water were available *ad libitum*, except during glucose tolerance test (GTT). The body weight was measured at the beginning of the study (at the age of 19 weeks), and after each chronic stress session (at the age of 20, 24, and 28 weeks).

### Ovariectomy

Female rats (n = 16, OVX group) were ovariectomized at the age of 12 weeks according to Harlan Laboratories protocol (29). Before this study, we performed two pilot studies showing that if ovariectomy was performed 4 weeks before animals were included in the stress protocol (before moving them to experimental room), stress caused by surgical procedure was irrelevant and fully compensated (data not shown). This allowed us not to use sham operated group and reduce the total number of animals used. In pilot studies, OVX and NON-OVX animals did not differ in behavioral response to handling before any stress was induced, moreover OVX animals were not more agitated. During overiectomy the animals were anesthetized with isoflurane (Forane® isofluranum, Abbott Laboratories Ltd, Queenborough, UK). Postoperatively animals were provided with food and tap water *ad libitum* and were closely monitored for 72 hours.

### Chronic stress protocol

**Figure 2:**
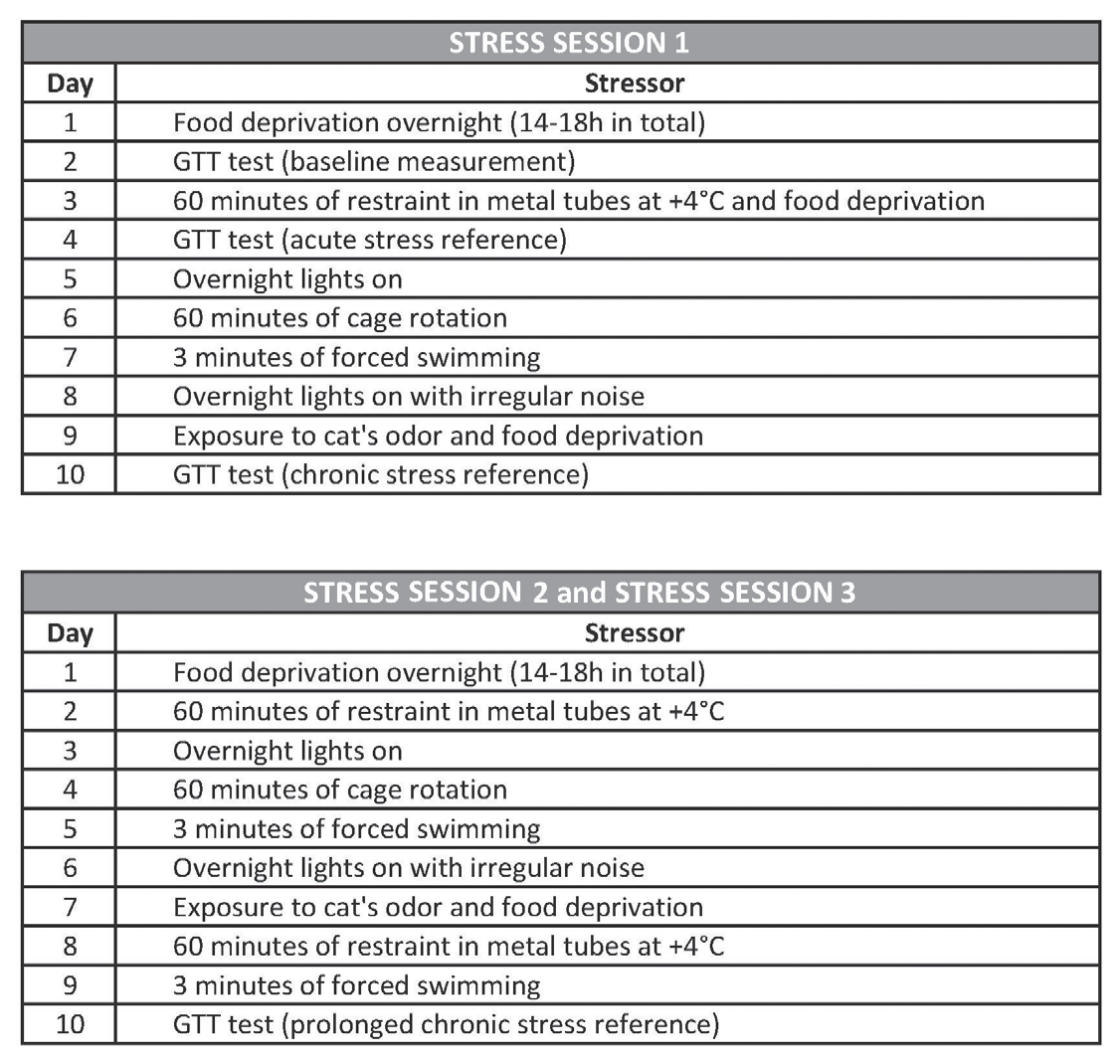
Detailed daily chronic stress protocol.

At 16 weeks age, all animals were moved from animal facility to animal experimental laboratory. Twenty four of them (8 males, 8 OVX, and 8 NON-OVX rats) were submitted to chronic stress at the age of 19 weeks (ie, 18 days later). Stress sessions (10-days of stress) were repeated three times. Stressors in the second and third stress session were uniformly performed in the same order and at the same time of the day. During stress session one, 3 GTT tests were performed (Figure 2) There was an 18-day period between the sessions and the protocol was finished when animals reached the age of 28 weeks. One of the stressors included exposure to cat’s odor, which caused immediate and a very strong stress for rats causing their bristles to visibly protrude. The cat was examined by veterinarian and declared as healthy to participate in the protocol. The rats were sacrificed at the age of 28 weeks and tissues, organs, and blood were collected.

### Sham stress protocol

The control group (6 males, 8 OVX, and 8 NON-OVX rats) was submitted to sham stress. They were exposed to the same environment and handling as the chronic stress group but the stressors were omitted. Protocol (10-days sham stress session) was repeated 3 times until animals reached the age of 28 weeks, when they were sacrificed and tissues and organs were collected as well as blood, which was drawn from the heart for serum measurements.

### Glucose tolerance test

GTT was an additional stressor and it was performed in chronic stress group only. GTT was performed 5 times: a day before the first stress session (baseline reference), a day after the first stressor (acute stress reference – data not shown), and after the first, second, and third 10-day session (references of prolonged chronic stress). The animals fasted overnight for 14-18 hours before measurement and this was considered as additional stressor for all chronic stress groups. First, 2 g/kg of glucose per body weight was injected intraperitoneally. Glucose concentration was measured at six time points – 0, 15, 30, 45, 60, and 90 minutes by OneTouch® UltraMini® Glucose Meter (Milpitas, CA, USA). The strength of a physiological factor can be measured by calculating the area under the curve (AUC) (30), which we applied for glucose tolerance test. AUCs were calculated using trapezoidal integration.

### Biochemical analyses

For biochemical analysis of glucose and cholesterol, serum was collected at the time of sacrifice. All animals were sacrificed during the daytime, two days after the last GTT was performed. After the last GTT, animals had the food available *ad libidum*, however, since rats do not feed during daytime (sacrificing was performed at 12-6 h pm) we assume that these measurements were performed pre-prandial. Upon deep anesthesia, blood was collected with a syringe from the right ventricle and transferred to 6 mL EDTA tubes. Serum was separated by 5 minutes of centrifugation at 3000 × g. Serum glucose concentration was measured using an enzymatic UV-assay (Cat. No: 05168791 190, Roche Diagnostics GmbH, Mannheim, Germany). The inter-assay coefficient of variation (CV) was <2% (lower detection limit: 0.85 mmol/L, upper detection limit: 45 mmol/L). Total cholesterol concentration was measured using a colorimetric assay (Cat. No: 05168538 190, Roche Diagnostics GmbH). The inter-assay CV was <2% (lower detection limit: 0.1 mmol/L, upper detection limit: 20.7 mmol/L). All measurements were performed on the Roche/Hitachi Cobas C 701 analyzer (Roche Diagnostics GmbH).

### Adrenal gland isolation

Animals were anesthetized using isoflurane (Forane® isofluranum, Abbott Laboratories Ltd) as inhalation gas in a glass chamber and intramuscular injection of Ketamine (Ketanest, Pfizer Corporation, New York City, NY, USA) at a concentration of 30 mg/kg. At the time of sacrifice both adrenal glands from each animal were isolated, fixed overnight in 4% paraformaldehyde in PBS, cryoprotected in 10%, 20%, and 30% sucrose (24 hours each) and snap frozen in pre-chilled isopentane. The samples were stored at -80°C for further analysis.

### Immunohistochemistry

For adrenal gland immunohistochemistry, 25-µm cryosections were used. After 1% H_2_O_2_ in PBS pretreatment for inactivation of endogenous peroxidase, sections were placed in blocking solution containing 1% bovine serum albumin and 5% goat serum in PBS for two hours at +4°C with gentle shaking. After blocking, the unspecific binding sections were incubated with primary antibodies up to 2 nights at +4°C. Primary antibodies were prepared in blocking solution and used in different dilutions: anti-ERβ (1:100), anti-PR (1:100), anti-AR (1:250), and anti-Ob-R (1:30) (Santa Cruz Biotechnology, Dallas, TX, USA). The sections were washed in PBS and incubated with secondary biotinylated goat anti-rabbit antibody (Jackson Immuno Research, West Grove, PA, USA) for 2 h at +4°C. After washing sections in PBS, secondary antibody was detected with Vectastain ABC kit (Vector Laboratories Inc., Burlingame, CA, USA) by incubation for 1 h. Sections were washed in PBS and visualized with peroxidase substrate kit (DAB) (Vector Laboratories Inc., Burlingame, CA, USA). Sections were mounted on slides, air-dried and scanned with Nikon Super CoolScan 9000 ED (Nikon Inc., Melville, NY, USA), coverslipped with Vectamount (Vector Laboratories Inc.) and imaged on Zeiss Axioskop 2 MOT microscope (Carl Zeiss Microscopy, Thornwood, NY, USA), with Olympus D70 camera (Olympus, Hamburg, Germany). Figures were assembled, adjusted for contrast, intensity and brightness in Photoshop (Adobe Systems Incorporated, San Jose, CA, USA) to remove signal background that occurred in control reactions. ERβ, PR, AR, and Ob-R were quantified by counting the cells that gave positive signal using free ImageJ software (US National Institutes of Health, Bethesda, MD, USA) in a field 200 × 200 µm under 400 × magnifications for each layer of adrenal gland.

### Statistical analysis

Distribution of data (weight, serum concentrations of glucose and cholesterol) was determined by Shapiro-Wilk test. For data with non-normal distribution, Kruskal-Wallis and Mann-Whitney were used. For data with normal distribution, *t* test for independent samples was used. Statistical significance level was defined as *P* < 0.050. Statistical tests were performed using the statistical software package SPSS (SPSS Inc. Released 2008. SPSS Statistics for Windows, Version 13.0, Chicago, IL, USA).

## Results

### The effect of chronic stress on body weight, biochemical parameters, and glucose tolerance test

**Figure 3:**
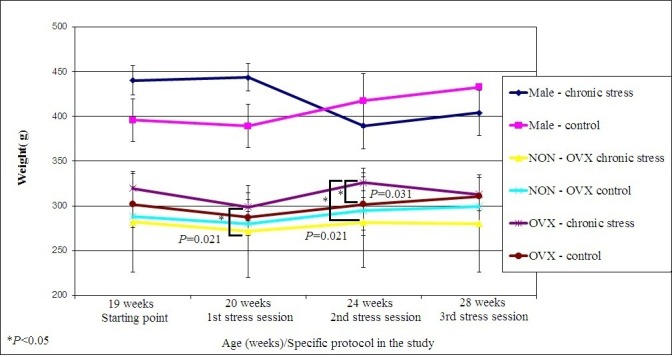
Body weights of male, ovariectomized (OVX), and non ovariectomized (NON-OVX) animal groups upon no stress (control) and chronic stress at the beginning of the study and after each stress session had started (weeks 19, 20, 24, and 28). Statistical significance level was set to *P* < 0.05.

Statistical analysis regarding the body weight was performed in all groups (Figure 3). Male chronic stress group showed overall loss of weight and specifically after the second round of chronic stress It had been shown that repeated stress has long term negative effect on body weight in male Sprague Dawley rats (31) and we expected similar finding. Weight in OVX chronic stress group was significantly higher than in OVX control group during the second stress session at week 24 (*P* = 0.031), and in NON-OVX group after the first (*P* = 0.021) and second (*P* = 0.021) stress session (weeks 20 and 24). NON-OVX chronic stress group had almost the same weight at the beginning and at the end of the study. Animal weights corresponded to physiologically expected values (32). Observed differences in weight between males and females during chronic stress were previously reported (33), and they could be considered a proof for validity of the chronic stress protocol.

### Both glucose and cholesterol in serum were lower upon chronic stress in ovariectomized rats

Serum measurements of cholesterol and glucose (Table 1) were performed in control and chronic stress groups not challenged with glucose for 24 hours. In OVX chronic stress group glucose concentrations were significantly lower than in control group (*P* = 0.001). Chronic stress resulted in a significant decrease in cholesterol levels in both female groups (*P* = 0.011 for OVX and *P* = 0.028 for NON-OVX) compared to control groups.

**Table 1:** Values of cholesterol and glucose concentration measured at the end of the treatment in male, ovariectomized (OVX), and non ovariectomized (NON-OVX) animal groups upon no stress (control) and chronic stress. Statistical significance level was set to *P* < 0.05.

	Male		OVX		NON-OVX	
	Control	Chronic stress	Control	Chronic stress	Control	Chronic stress
**Glucose (mmol/L),** mean ± standard deviation	8.45 (±1.38)	7.8 (±1.33)	8.71 (±2.84)	4.87 (±2.38)	7.78 (±0.71)	9.37 (±1.17)
			*P = 0.001			
**Cholesterol (mmol/L),** mean ± standard deviation	1.86 (±0.21)	1.67 (±0.18)	2.79 (±0.39)	2.32 (±0.34)	2.61 (±0.29)	2.34 (±0.92)
			*P = 0.011		*P = 0.028	

### GTT results were affected upon chronic stress

**Figure 4:**
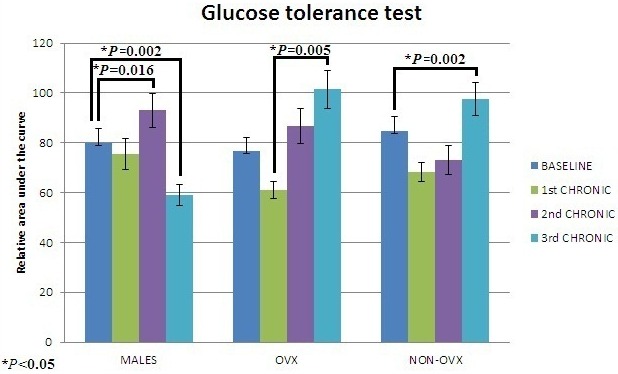
Quantification of glucose tolerance test relative areas under the curve (AUC) of male, ovariectomized (OVX), and non ovariectomized (NON-OVX) animal groups upon no stress (baseline) and three sessions of chronic stress. Statistical significance level was set to *P* < 0.05.

The stress influenced the GTT results. Male and OVX group showed similar results at all the time points. Males showed significantly higher AUCs after the second stress session (*P* = 0.016) and significantly lower AUCs after the third stress session (*P* = 0.002) in comparison to baseline measurement. In NON-OVX group, the third chronic stress session significantly increased glucose levels compared to baseline measurement (*P* = 0.002). In OVX group, AUC value was significantly higher after the third stress session than after the first stress session (*P* = 0.005) (Figure 4).

### Sex-specific differences in expression of sexual hormone receptors and leptin receptor in adrenal gland upon chronic stress

Four zones of adrenal gland were taken into account: *zona glomerulosa, zona fasciculata, zona reticularis, and medulla.* Significant changes were observed in *zona glomerulosa* and *zona reticularis* so in further presentation we focused on these layers only (Table 2).

**Table 2:** Values of sexual hormone and leptin receptors immunopositive cells in the *zona glomerulosa* (for estrogen receptor beta and progesterone receptor) and *zona reticularis* (for testosterone and leptin receptors) in ovariectomized (OVX), non ovariectomized (NON-OVX), and male control and chronic stress groups. Statistical significance level was set to *P* < 0.05.

	Male Mean (±SD) (stained cell count/0.04mm^2^)	OVX Mean (±SD) (stained cell count/0.04mm^2^)	NON-OVX Mean (±SD) (stained cell count/0.04mm^2^)
**Estrogen receptor beta in *zona glomerulosa***			
**Control**	2.3 (±2.08)*	3.7 (±1.53)	104.3 (±41.66)
**Chronic stress**	23 (±10.44)*	12.7 (±5.50)	57.7 (±21.96)
**Progesterone receptor in *zona glomerulosa***			
**Control**	10 (±.30)	15.3 (±6.80)	21 (±5.2)
**Chronic stress**	4 (±2)	4 (±1)	11.3 (±5.5)
**Testosterone receptor in *zona reticularis***			
**Control**	28.3 (±8.5)	79.6 (±5.50)	84 (±12.28)^†^
**Chronic stress**	35.6 (±7.23)	204.3 (±74.89)	32.3 (±12.42)^†^
**Leptin receptor in *zona reticularis***			
**Control**	6 (±3.46)	7.3 (±2.30)^‡^	8 (±2)
**Chronic stress**	14 (±6. 1)	14.6 (±3.21)^‡^	16.3 (±6.80)

*P = 0.028.

†*P* = 0.007.

‡*P* = 0.033.

**Figure 5:**
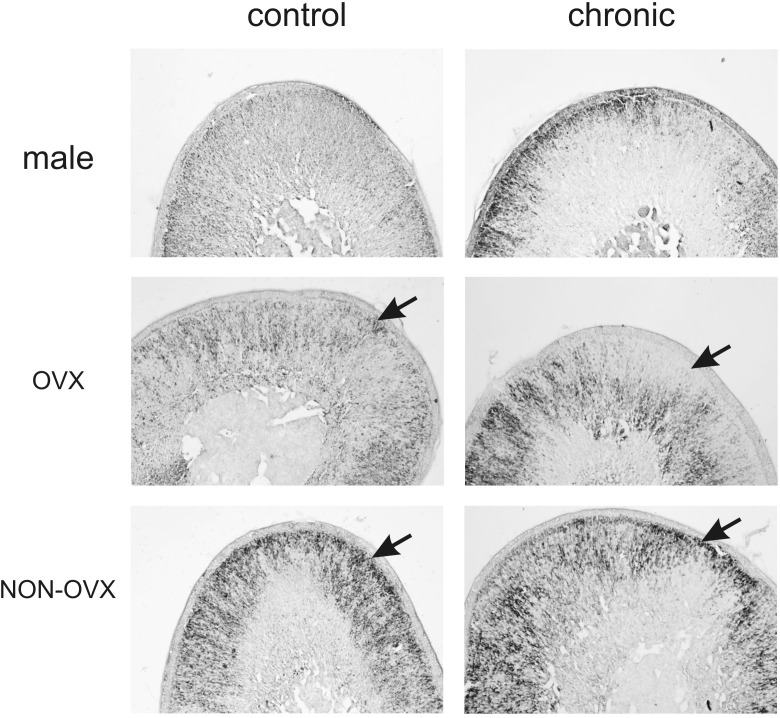
Ob-R antibody immunostaining of the adrenal gland (25 µm sections) *zona reticularis* in male (**M**), ovariectomized (OVX), and non-ovariectomized (NON-OVX) group upon no stress (control) and upon chronic stress. Arrows indicate the presence of strong nuclear staining. Pictures were taken under 400 magnification.

Male and OVX group had the same low basal levels of ERβ receptors in *zona glomerulosa*. In male chronic stress group, the number of ERβ receptors significantly increased compared to male control group (*P* = 0.028). In male and OVX group, the values of AR in *zona reticularis* increased upon chronic stress. In NON-OVX chronic stress group AR positive cells significantly decreased (*P* = 0.007). The levels of PR in *zona glomerulosa* did not change in any consistent pattern. The levels of Ob-R in *zona reticularis* increased in all groups (Figure 5), but the significant difference was reached only in OVX chronic stress group compared to OVX control group (*P* = 0.033).

## Discussion

This study showed changes in cardiovascular risk factors and a set of receptors in the adrenal gland using a model of chronic stress in rats (34). Human studies on obesity that separate sex-specific results usually report the same phenomenon; chronic stress is not a risk factor for developing obesity in men (35). On the contrary, it is a risk factor for developing obesity in women (36). In our study, male chronic stress group showed overall loss of weight, while NON-OVX chronic stress group showed no weight gain toward the end of the study. This result suggests a good feedback system of body weight maintenance. Concluding from OVX females, maintenance of body weight did not depend on the ovarian function itself, but possibly on overall compensatory production of different hormones, including sexual hormones produced by the adrenal gland.

Classical clinical markers of cardiometabolic risk (dyslipidemia, metabolism of glucose, and cholesterol) are good candidates to verify the influence of stress (13,14,37). In this study both serum glucose and cholesterol were lower upon chronic stress, in particular in OVX group. Data about cholesterol levels upon chronic stress in humans are ambiguous – some studies report decrease (38), but more studies report elevation (39). We could assume that the observed low measurements of glucose and cholesterol were a sign of stress compensation during peak of reproductive age (our animals were considered to match 20-30 old humans).

Peritoneal GTT is considered as valuable orientation sign of glucose metabolism changes in animal studies (40). Impaired glucose tolerance after surgical removal of ovaries in premenopausal women (due to treatment of cancer) is reported in almost half of the patients after 12 months period (41). In our study males and OVX animals showed similar AUCs trend with the worst glucose tolerance after the second stress measurement and increased tolerance after the third. This could be explained by the young age of the animals and their assimilation to stress or the onset of hyperinsulinemia. The similarity of OVX and male groups could be explained by similar physiology due to the lack of sexual hormones excreted by the ovaries.

Estrogen, testosterone, and progesterone receptors are autoregulated and have been identified in the adrenal gland of many animals, including rodents (42-44). Moreover, overlap of estrogen, progesterone, and testosterone receptors expression is observed in the neurons of paraventricular nuclei (PVN) in humans and animals (45,46).

ERβ in adrenal gland were involved in maintenance of homeostasis in non-ovariectomized females. Stress induced up-regulation of testosterone receptor in adrenal glands in ovariectomized females and males may implicate change in testosterone regulation via the HPA axis. Since the production of testosterone in OVX females can only occur in the adrenal gland (47), this finding could mean that besides cortisol, testosterone was also involved in regulation of overall secretion by *zona reticularis* cells in OVX females. Progesterone receptor was not influenced by chronic stress, while Ob-R was strongly up-regulated in ovariectomized rats. Besides its effect on body weight regulation, leptin also suppresses the HPA through its negative effect on corticotropin-releasing hormone (CRH) in response to stress (48). Ob-R antibody staining was analyzed in the *zona reticularis,* which is responsible for steroid hormones production (49). The strong receptor expression was noticed in all animal groups after chronic stress. The leptin receptor signaling pathway seemed to be especially important for endocrine cells, which are producing reproductive hormones.

In conclusion, body weight, GTT changes, and specific pattern of biochemical parameters changed in response to stress and were a useful tool for validating chronic stress protocol. Some exceptions in measurements could be explained by the young age of the animals. In response to stress, early changes in sex specific pattern were detected in the adrenal gland. Changes upon chronic stress in the adrenal gland were related to a decrease in testosterone receptor in females and increase in estrogen receptor in males.
